# Effects of Three-Dimensional Graphene–Carbon Nanotube Hybrid on the Mechanical Properties and Microstructure of Cement Paste

**DOI:** 10.3390/ma16196571

**Published:** 2023-10-06

**Authors:** Xin Zhao, Li Qiu, Deyu Kong, Yangfei Huang, Jintao Liu

**Affiliations:** 1 Department Disaster Mitigation Structure, College of Civil Engineering, Tongji University, Shanghai 200092, China; xinzhao@zust.edu.cn; 2 College of Civil Engineering and Architecture, Zhejiang University of Science and Technology, Hangzhou 310018, China; 3 China Railway Eryuan Engineering Group, East China Survey and Design Co., Ltd., Hangzhou 310009, China; moonxiaoshu@126.com; 4 College of Civil Engineering, Zhejiang University of Technology, Hangzhou 310018, China; kongdeyu@zjut.edu.cn

**Keywords:** graphene, carbon nanotubes, microhardness, microstructure, hydration

## Abstract

This work experimentally studies the mechanical properties and microstructure of cementitious composites reinforced with a three-dimensional graphene–carbon nanotube (CNT) hybrid. Firstly, the graphene–CNT (GC) hybrid is dispersed in cement pastes using ultrasonication and surfactant, and then, the effect of the GC hybrid on the early hydration of the cement pastes is investigated. The experimental results show that adding the GC hybrid shortens the setting stage of cement hydration and accelerates the early hydration process. Moreover, the macro- and micro-mechanical properties of each group are evaluated. The 7- and 28-day strength of the cement pastes improves with addition of the GC hybrid. Finally, the microstructural analysis demonstrates that the GC hybrid is reasonably well distributed in cement and forms a spatial network, which could bridge the cracks and compact the cementitious matrix.

## 1. Introduction

Cementitious materials have been widely used for construction, but their brittleness restrains their development and further use. Although traditional fibers can restrain crack propagation on a macro scale, they are unacceptable for delaying the initiation of microcracks [1]. Due to their unique mechanical properties and potential applications in building materials, nanomaterials have received tremendous attention in recent years. According to their morphology, nanomaterials are usually categorized into zero-dimensional (0-D), one-dimensional (1-D), and two-dimensional (2-D). Zero-dimensional nanomaterials, such as nanoparticles, are usually used as nanofillers and cannot restrain nanosized cracks. One- and two-dimensional nanomaterials, such as carbon nanotubes (CNTs) and graphene, enjoy many advantages in modifying the performance of cementitious composites [2,3].

As one-dimensional fibers, CNTs are allotropes of carbon with a cylindrical nanostructure and a tensile strength of 63 GPa [4]. Thus, CNTs have been widely studied for enhancing the toughness of cementitious materials [1]. Li et al. [5] added 0.5 wt% (by weight of cement) CNTs to mortar and found that its flexural and compressive strength increased by 18.86% and 25.11%, respectively. Gdoutos et al. [6] showed that the addition of CNTs to plain mortar enlarged the flexural strength by 86.7% and the critical stress intensity factor/fracture toughness by 85.7%. Furthermore, CNTs can improve the nano-mechanical properties of cement paste and increase the content of the high-stiffness calcium silicate hydrate (C-S-H) gel [7,8]. Regarding the microstructure, CNTs provide a more extensive interface for stress transfer, limit the growth of nanosized cracks, and hinder the formation of micropores [9]. Previous studies have shown that the addition of 0.1–0.5 wt% (by weight of cement) CNTs resulted in a 200% increase in the volume fraction of high-density C-S-H [10], and CNTs had a greater effect upon the overall nanocomposite hydration and microstructural development [11]. However, CNTs are prone to forming agglomerates due to their high aspect ratio and van der Waals interactions. Unlike CNTs, graphene comprises a single-layer sheet of carbon atoms closely packed into a 2-D honeycomb framework [12]. The Young’s modulus of graphene reaches 1.0 TPa, and its tensile strength is in excess of 130 GPa [13]. Because of their particular structure, graphene sheets can regulate the formation of flower-like (AFt, AFm, and CH) and polyhedron crystals and compact the microstructure of cement paste [14,15]. Peng et al. [16] indicated that adding graphene improved the toughness of cement composites, and the increase in flexural strength was more significant than that in the compressive strength. Liu et al. [17] indicated that the introduction of graphene enhanced the mechanical properties of cement composites, and adding 0.05% (by weight of cement) of graphene increased the fracture energy of the composites by 37%. Analyzing the microstructure revealed that hydration products were connected by graphene, and graphene could bridge the cracks in the cementitious matrix [18,19,20].

Theoretically, combining CNTs and graphene may help to improve the interaction bonding and transfer more load from the cementitious matrix to nanoadditives [21]. Zhou et al. [22] used graphene sheets as a surfactant to disperse CNTs in water, and the addition of a hybrid of graphene oxide (GO) and CNTs increased the fracture resistance of the composites. Another study reported that the flexural and compressive strength of the cement paste remarkably increased by 21.13% and 24.21%, respectively, with the incorporation of the GO–CNT hybrid [23]. However, studies on the combined effects of graphene and CNTs on the performance of cementitious materials are still scarce [24].

Thus, this paper proposes a new type of nanomaterial, i.e., a three-dimensional graphene–CNT (GC) hybrid, and explores its effect on the mechanical properties of cement pastes. Different concentrations of the GC hybrid (0.00%, 0.05%, 0.10%, and 0.15% by weight of cement) were added to the cement, and the compressive and flexural strength of the composites were evaluated. Further, electrical resistivity tests examined the early hydration process of the composites. The microstructure of the composites was also analyzed utilizing microhardness, scanning electron microscopy (SEM), and X-ray diffraction (XRD).

## 2. Experimental Procedures

### 2.1. Materials

**Figure 1:**
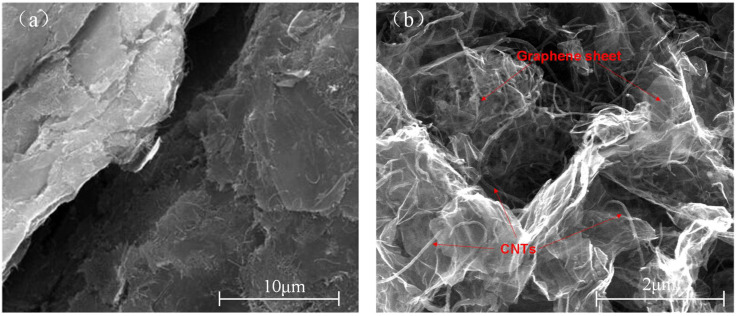
The SEM micrograph of the GC hybrid with low magnification (**a**) and high magnification (**b**).

**Figure 2:**
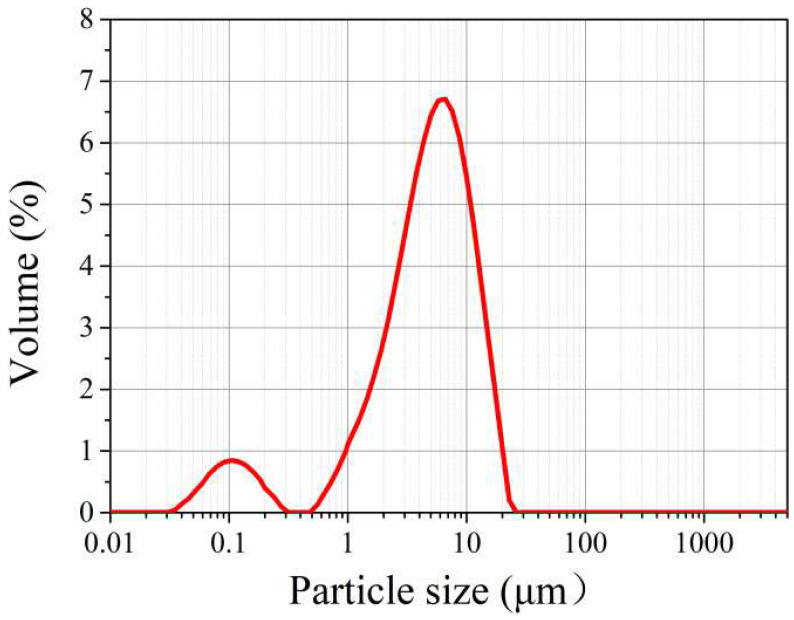
The particle size distribution of the GC hybrid.

In this study, Ordinary Portland Cement (PO 42.5, Type II) was used. Chengdu Organic Chemicals Co., Ltd. (Chengdu, China), provided the GC hybrid with the properties in Table 1. The composition proportion of graphene sheets and CNTs was 4:1. Figure 1 and Figure 2 illustrate the SEM image and particle size distribution of the GC hybrid. In this study, the diameters of most GC were mostly distributed from 16 µm to 20 µm, and the median size of GC was about 3 µm to 6 µm. On the basis of previous studies [9,14,17], a high-efficiency nonionic surfactant with an active content of 90% and a cloud point of 68–70 °C, purchased from Chengdu Organic Chemicals Co. Ltd. (Chengdu, China), was used for dispersing the GC hybrid in cement.

### 2.2. Dispersion of GC Hybrid

Previous studies have shown that graphene and CNTs tend to form aggregates due to solid intermolecular van der Waals interactions. The key to increasing the performance of the composites based on the GC hybrid is improving the GC dispersity in the cementitious matrix. Thus, this work used a surfactant combined with an ultrasonic instrument. The structure of the surfactant consisted of hydrophilic groups and aromatic rings, which could improve the dispersibility of the GC hybrid in water. Firstly, the GC hybrid powder and surfactant were added to water and stirred evenly. Then, an FS-750T ultrasonic cell disrupter (20 kHz, 600 W) from Shanghai Sonxi Ultrasonic Instrument Co., Ltd (Shanghai, China)was employed to disperse the GC hybrid in water for 30 min. The suspension was placed in ice water during dispersion to prevent heating and foaming caused by the sonication process, and the ice-water temperature was kept below 5 degrees.

**Figure 3:**
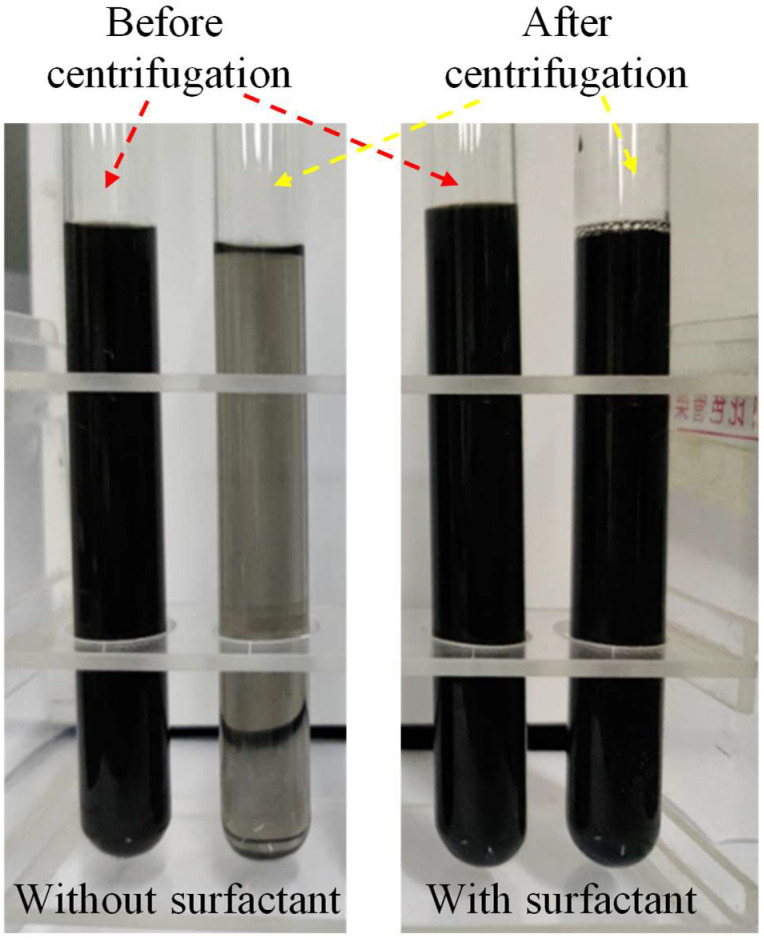
The images of the GC hybrid dispersions before and after centrifugation.

Sedimentation tests were conducted on the GC hybrid dispersions using a TD80-1 model centrifugal machine (Xicheng Xinrui Instrument Factory, Changzhou, China) to evaluate their long-term stability. The centrifugal force generated by the centrifuge is much larger than its gravity, which better reflects the stability of the GC hybrid dispersions. The centrifuge speed was set at 2000 rpm (1435 g), and the centrifugation period was 60 min. Figure 3 displays the images of the GC hybrid dispersions before and after centrifugation. The experimental results indicate the excellent long-term stability of the GC hybrid dispersion.

### 2.3. Preparation of Specimens

As shown in Table 2, the weight ratios of water to cement and surfactant to cement were kept at 0.4 and 0.02%, respectively. According to the ASTM standard (ASTM C305-06 [25], Standard practice for mechanical mixing of hydraulic cement pastes and mortars of plastic consistency), the weighed cement was put into a JJ-5 mixer during the mixing process. Then, the preparative GC hybrid dispersion was poured into the mixer slowly and stirred for 180 s at a low speed of 140 rpm. Finally, the mixture was stirred at a high speed of 285 rpm for 120 s to further disperse the GC hybrid.

According to GB/T17671-1999 (National Standard of China) [26], the fresh cement pastes were poured into molds with the dimensions 40 mm × 40 mm × 40 mm cubic and 40 mm × 40 mm × 160 mm prismatic for the compressive and flexural tests, respectively. After the cement pastes were poured into molds, the specimens were cured in a standard curing environment (20 ± 1 °C, 95%RH) for 24 h, and then, the specimens were removed from the molds. They were maintained in a curing room at a temperature of 20 ± 1 °C and relative humidity higher than 95% until testing. 

### 2.4. Electrical Resistivity

**Figure 4:**
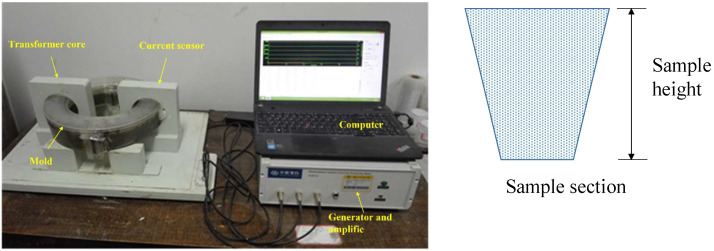
The noncontact resistivity measurement system and trapezoidal cross-section of the specimen.

In order to study the influence of the GC hybrid on the early hydration of cement, we monitored the electrical resistivity of the mixtures using an electrodeless fresh cement resistivity analyzer (CCR-3, Hong Kong Brilliant Concept Technologies, Hong Kong, China). The principle of the measurement was given by Wei and Li [27,28]. The electrical resistivity of the cement paste was measured by a non-contact electrical resistivity apparatus, and there was no electrode inserted into the paste, as shown in Figure 4. Each sample was cast into the mold (1672 cm^3^ in volume) to a specific height and sealed with plastic covers, and a trapezoidal cross-section specimen is shown in Figure 4. The height of the specimen was generally controlled at 40 mm. The measuring equipment was in a temperature-controlled room with a constant temperature of 20 °C. The resistivity development of the cement pastes was recorded for 3 days, as shown in Figure 4.

### 2.5. Mechanical Tests

According to the Chinese standard “Method of Testing Cements: Determination of Strength” (GB/T 17671-1999) [26], the compressive strength and flexural strength of the composites were investigated at a curing age of 3, 7, and 28 days. During testing, the loading rate was also set at 2.4 kN/s and 50 N/s, respectively.

In order to evaluate the influence of GC on the micromechanical properties of the GC composites, a commercially available microhardness tester was used. A Vickers microhardness tester (402 MVD, Wilson Instruments, IL, USA) was used to measure the hardness of the samples. After 28 days of curing, a small piece of the cement samples (5 mm × 5 mm × 5 mm) was first embedded in a rubber powder by a pointing machine. Then, the prepared samples were polished with 125, 65, 38, 15, 10, and 6.5 µm metallographic sandpapers, respectively, using a grinder polisher until the surface was adequately smooth and flat. Anhydrous ethanol was used as a cooling lubricant in the polishing process. According to ASTM E384-99 [29], 100 points in a 10 × 10 grid were measured on the surface of each sample within a distance of 150 µm for the hardness. The loading force was 50 gf (490 mN), and the loading period was 10 s. The microhardness of composites was distributed between 20 and 120 MPa. According to the previous literature [30], the probability of microhardness was calculate at 10 MPa intervals, and the probability plot of composites was drawn based on polynomial curves.

### 2.6. Microstructure Characterization

Fragmented pieces from the mechanical test were saved for microscopic characterization, and the specimens were vacuum-dried at 60 °C for 48 h before testing. Mercury intrusion porosimetry (MIP) was employed to examine the pore structure and size distribution of the cement pastes. The analyses were conducted using a Micromeritics AutoPore IV 9500 (Micromeritics Instrument Corporation, Norcross, GA, USA), which could theoretically determine a pore diameter of up to 3 nm under high pressure. Moreover, the samples were coated with gold for the SEM analysis using an FEI Quanta 650 scanning electron microscope, and EDS was also used to analyze the element composition. A Bruker D8 Advance X-ray diffractometer (Bruker Corporation, Billerica, MA, USA) with monochromatic CuKa radiation was employed to identify the hydration products.

## 3. Results

### 3.1. Compressive and Flexural Strength

**Figure 5:**
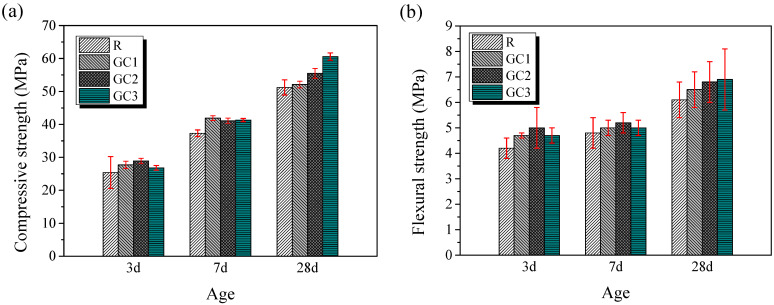
The compressive (**a**) and flexural (**b**) strength of the cement pastes cured for 3, 7, and 28 days.

The compressive and flexural strength of the specimens was measured at a curing period of 3, 7, and 28 days. Figure 5 depicts the average strength of the samples. The strength of the mixtures containing the GC hybrid improved at different curing ages. At a curing period of 7 days, the average compressive strength of specimens GC1, GC2, and GC3 improved by 12.3%, 10.2%, and 10.7%, respectively, compared with the blank. Similarly, the flexural strength of specimens GC1, GC2, and GC3 increased by 4.2%, 8.3%, and 4.2%, respectively. Furthermore, the enhancement effect of the GC hybrid improved with the curing age. Notably, the compressive and flexural strengths of the composite with 0.1 wt% of the GC hybrid were higher than those of the blank by 18.4% and 13.1%, respectively, at a curing period of 28 days.

### 3.2. Electrical Resistivity

**Figure 6:**
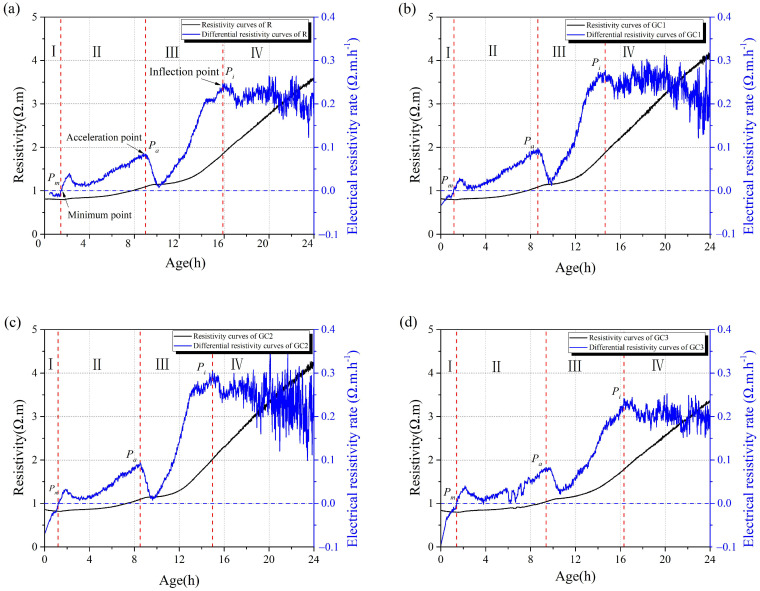
The resistivity and differential resistivity curves of the cementitious composites: (**a**) the blank; (**b**) specimen GC1; (**c**) specimen GC2; (**d**) specimen GC3.

The resistivity of the cement paste changes dramatically in the early hydration stages, and it usually reflects the variation in the ion concentration and pore structure during cement hydration. Figure 6 delineates the electrodeless resistivity and differential resistivity curves of the composites. According to our previous study [31], we divided the resistivity curves into four stages, Ⅰ, II, III, and IV, using three critical points: *P_m_*, *P_a_*, and *P_i_*.

The *dissolution stage* (Stage I) is from the starting point to the minimum point (*P_m_*) on the electrical resistivity curve. In this period, the dissolution of cement particles increases the ion concentration, reducing the electrical resistivity of the cementitious composites. Compared with the control group, adding the GC hybrid shortened Stage I, which might be related to the high surface energy of the GC hybrid.

The *setting stage* (Stage II) is from point *P_m_* to the acceleration point (*P_a_*) corresponding to the second peak point on the differential curve. In this period, the initial cement hydration reaction consumes some ions, and the resistivity of the sample increases a little. The early hydration products, such as the C-S-H gel and calcium hydroxide (Ca(OH)_2_), are gradually formed. The resistivity of the cementitious composites increases slowly, and the cementitious mixtures stiffen and gain strength. As shown in Figure 5, the setting stage was accelerated with the addition of the GC hybrid. Previous studies have reported that graphene sheets with a high specific surface area have a seeding effect on cement hydration, providing abundant additional sites for the nucleation and growth of hydration products [32,33].

The *acceleration stage* (Stage III) is from point *P_a_* to the third peak point (*P_i_*), corresponding to the maximum point on the differential curve. In this stage, the electrical resistivity of the mixture increased quickly because of the rapid growth of the cement hydration products.

The *deceleration stage* (Stage IV): After point *P_i_*, the electrical resistivity of the mixture increases continuously, but its growth rate drops gradually. In this stage, the cement paste hydrates and hardens continually as the curing period extends, and the strength of the cement paste improves. It should be noted that the electrical resistivity of specimen GC3 was lower than that of specimen R at a curing period of 24 h, which was due to the high conductivity of the added GC hybrid.

**Figure 7:**
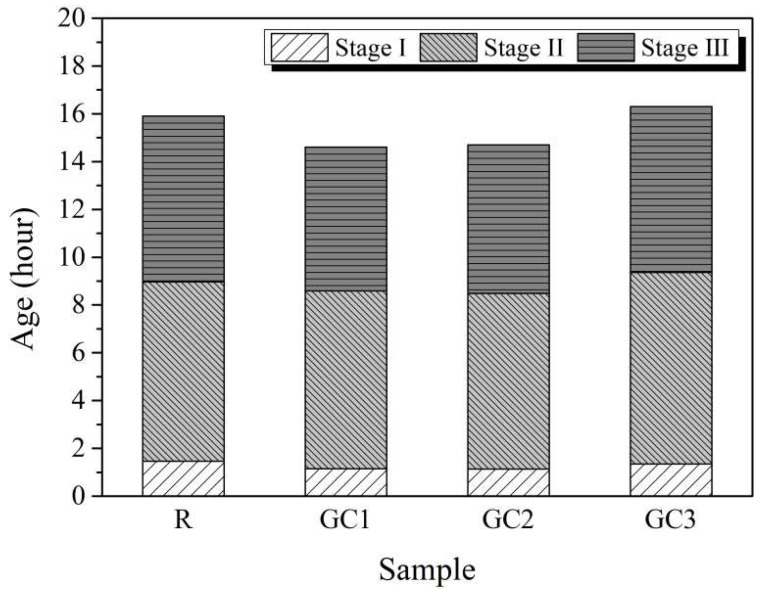
The duration of Stages I, II, and III.

Figure 7 plots the durations of Stages I, II, and III for each group. Adding the GC hybrid shortened the duration of the three stages. The duration of Stages I, II, and III was 1.5, 9.0, and 15.9 h, respectively, for the blank and 1.1, 8.5, and 14.7 h, respectively, for group GC2. However, the cement paste with 0.15 wt% of the GC hybrid (specimen GC3) did not significantly change compared with specimen R, which might be related to the agglomeration of the GC hybrid. The agglomerated particles impeded the growth of cement hydration products on the graphene sheets.

In general, the early-age resistivity development of the cement paste is inextricably linked to the strength [28]. From Figure 6, the resistivity of R group was about 3.5 Ω·m at 24 h, and the resistivity of specimens GC1 and GC2 improved by 17% and 20%, respectively, compared with the blank. This means that the addition of GC accelerated the early hydration process of the cement, and the early-age strength of composites showed a similar pattern to Figure 5. In addition, the R group and GC3 had roughly the same resistivity at 24 h, and they had similar strengths at 3 days.

### 3.3. XRD Analysis

**Figure 8:**
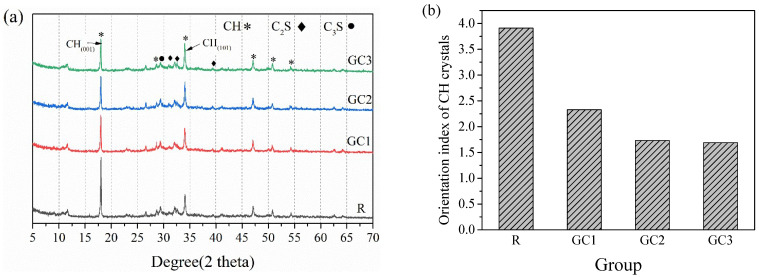
(**a**) The XRD analysis of the cementitious composites, and (**b**) the orientation index of the CH crystals at a curing period of 28 days.

Figure 8a depicts the crystal phases of the cement paste with different amounts of the GC hybrid at a curing period of 28 days examined using XRD. Portlandite (CH), as primary hydration products, was found in these samples, and tricalcium silicate (C_3_S) and dicalcium silicate (C_2_S) were also detected because of the unhydrated cement particles. According to Grandet and Ollivier [34], the orientation index (*R*) of the (001) crystal plane can be calculated as *R* = 1.35 × *I*_(001)_/*I*_(101)_, where *I*_(001)_ and *I*_(101)_ are the (001) and (101) crystal face peak intensities of the CH crystal, respectively. This index is related to the growth of the CH crystal during cement hydration, and the lower the orientation index, the higher the density of the microstructure.

Figure 8b depicts the influence of the GC hybrid on the CH orientation of the cement paste, and the orientation index dropped with an increase in the GC hybrid content. This result indicates that adding the GC hybrid enhances the growth of the CH, which was also reported by previous studies [35,36]. Graphene sheets and CNTs have a high specific surface area, strength, and flexibility, and the surface of the GC hybrid has many oxygen-containing functional groups. Thus, the cement hydration reactions tend to occur continuously on the surface of the graphene sheets and CNTs. Previous studies have demonstrated that graphene or CNTs with a larger specific surface will provide the nucleation site for the cement hydration reaction and eventually constrain the degree of the cement hydration reaction in the early stage [37]. The mechanical test results generally indicated that the strengths improved markedly with the addition of GC at 3 d and 7 d. the addition of GC had no distinct impact on the strength of the cement paste at 28 d, which explained why the reduction in peak intensities for C_2_S and C_3_S for the GC samples was not obvious at 28 days based on the XRD results.

### 3.4. Microhardness

**Figure 9:**
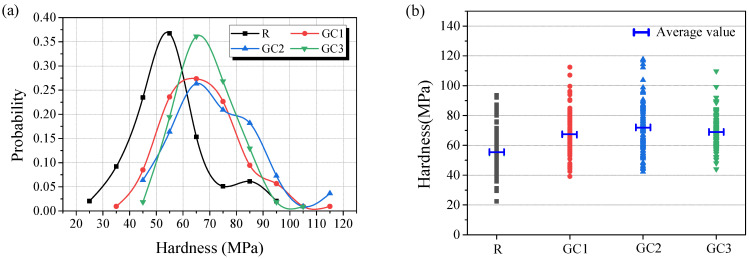
The probability plots (**a**) and average microhardness (**b**) of the cementitious composites at a curing period of 28 days.

There are unhydrated cement particles, different kinds of hydration products, and pores and defects in the sample; thus, the average hardness of specimens can only reflect the influence of GC on the cement paste to a certain extent. On this basis, the probability plot of the sample microhardness was used for the characterization of the enhancement of GC. Figure 9a delineates the probability plots of the microhardness of the cementitious composites, reflecting their microstructure and mechanical properties. The experimental results show that the hardness of the cement paste improved by incorporating the GC hybrid. The peak probability plot of the blank ranged from 35 to 65 MPa, and adding the GC hybrid made the peak shift to the aspect of high hardness. When the content of the GC hybrid was 0.1 wt%, the peak probability plot of specimen GC2 ranged from 55 to 85 MPa. As shown in Figure 9b, the average hardness of the samples incorporating the GC hybrid also improved significantly. Compared to the reference specimen, the average hardness of specimens GC1, GC2, and GC3 increased by 22.9%, 31.8%, and 25.5%, respectively. Due to its high specific surface area, the GC hybrid showed high interfacial adhesion to the hydration products, which resulted in its excellent enhancement effect on the microstructure of the cement paste.

### 3.5. Porosity and Pore Size Distribution

**Figure 10:**
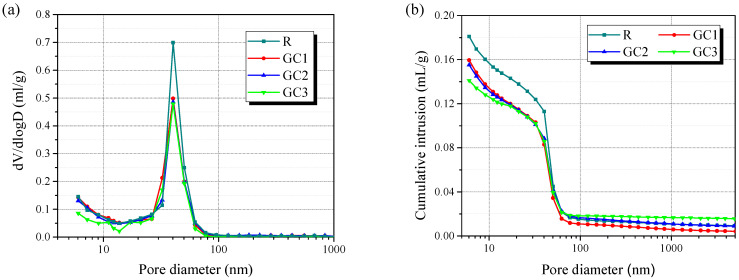
The pore size distribution (**a**) and cumulative intrusion (**b**) of the cementitious composites at a curing period of 28 days.

Analyzing the porosity of the GC hybrid–cement composites is vital since it correlates closely with their mechanical properties. The pore information of the cement pastes with different amounts of the GC hybrid was analyzed at a curing period of 28 days using MIP, as shown in Figure 10. Figure 10a presents the distribution of pore diameters of the cement pastes containing different amounts of the GC hybrid. The diameter of most pores ranged from 5 to 100 nm, and the average pore diameter of the control group was 24.3 nm. The average pore diameter of composites GC1, GC2, and GC3 was 22.0, 23.2, and 27.3 nm, respectively. Generally, the pores of cement pastes are divided into large capillary pores (10 to 0.05 µm), medium capillary pores (50 to 10 nm), and gel pores (<10 nm). It appears that the number of tiny pores (<50 nm) in the cementitious matrix declined significantly with the incorporation of the GC hybrid, which implies that the modification of the cement paste pore structure due to adding the GC hybrid was reflected by changes in the medium capillary pores and the gel pores.

Moreover, adding the GC hybrid to the cement pastes reduced their total porosity. The total porosity of the control group was 27.1%, and adding 0.05, 0.10, and 0.15 wt% of the GC hybrid reduced the total porosity by 24.3%, 23.9%, and 20.4%, respectively. The decreased gel porosity resulted from a higher hydration degree and the high surface activity and filling effect of the GC hybrid. Figure 10b presents the cumulative mercury intrusion measurements. The cumulative mercury intrusion declined by adding 0.05 and 0.10 wt% of the GC hybrid at the same pore diameter. However, the cumulative mercury intrusion of specimen GC3 was higher than that of the control group when the pore diameter was higher than 50 nm, which might be caused by the agglomeration of the GC hybrid. Due to the high van der Waals interactions between the GC hybrid sheets, they tended to form bundles and become defect sites in the cementitious matrix.

### 3.6. Microstructure

**Figure 11:**
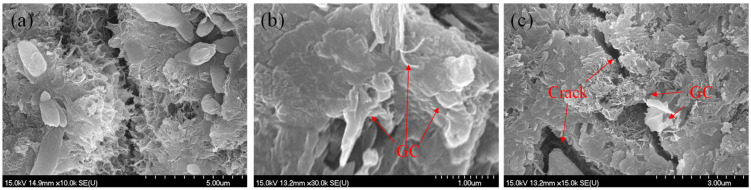
The SEM images of the GC hybrid–cement composites cured for 28 days: (**a**) specimen R; (**b**) specimen GC1; (**c**) specimen GC2.

Figure 11 illustrates the SEM images of the blank and the GC–cement composites at a curing period of 28 days. As shown in Figure 11a, the matrix of the reference specimen showed irregular needle-like crystals (Aft), and the matrix density was not high, allowing cracks to pass through the hydration products in a straight-through manner. The microstructure of specimens GC1 and GC2 was denser than that of group R, and needle- and rod-like crystals were reduced. Due to the high specific surface area of graphene, the GC hybrid could provide a platform for the C-S-H gel nucleation, as shown in Figure 11b. The hydration products of the specimens with the GC hybrid showed a more homogeneous microstructure than those of the blank.

Further, compact and small-sized C-S-H gels were observed, and little gel porosity was noticed. The GC hybrid comprised graphene and CNTs, both of which have a much higher tensile strength (over 100 GPa) than the cementitious matrix. Moreover, the unique three-dimensional structure of the GC hybrid increased the interactions between the cementitious matrix and the GC hybrid. The uniformly dispersed GC hybrid could also act as a bridge between pores and cracks (Figure 11c), significantly enhancing the mechanical properties of the GC hybrid–cement composites.

## 4. Discussion

This paper proposes a new type of nanomaterial, i.e., a three-dimensional graphene–CNT (GC) hybrid, and explores its effect on the mechanical properties of cement pastes. Although CNTs have excellent mechanical properties, they are prone to curling or agglomeration during the mixing. Moreover, Previous studies have shown that the addition of graphene can accelerate the cement hydration reaction as a result of the nucleation effect, and these hydration products might grow on it due to the large specific surface area. Theoretically, the three-dimensional GC hybrid might provide more effective enhancements of the cement paste. The GC hybrid has a favorable influence on the mechanical behavior of cement, mainly represented in two aspects: the early hydration and microstructure enhancement of the cement paste. It has been reported that graphene sheets can offer a higher surface area for the nucleation of CSH, which results in better interlocking between graphene and hydration products [38,39]. In this study, GC formed a net-like arrangement showing good incorporation into the cement matrix, and it increased load transfer efficiency between the matrix and GC. On the basis of the electrical resistivity analysis, the shortened duration of Stages I, II, and III could result from the accelerated transformation of the C-S-H gel surrounding the cement particles. Table 3 presents the enhancement effect of various carbon nanomaterials, i.e., multiwalled carbon nanotubes (MWCNTs), graphene sheets, and the GC hybrid, on cement paste, compared to our previous study. The GC hybrid has higher reinforcement and toughening properties at the same weight fraction. In particular, the average microhardness of the GC hybrid–cement composites is much higher than that of the CNT–cement or graphene–cement composites.

Strong covalent bonding at the interface between the GC hybrid and the hydration products deposited on the graphene sheets makes the GC hybrid a promising material for enhancing the cementitious matrix. The improvement in the microstructure of the cementitious composites is reflected by their mechanical properties (compressive strength) since adding the GC hybrid increases the micromechanical properties of the cement paste. This work used microhardness techniques to examine the difference in the micromechanical properties of the specimens. Moreover, the GC hybrid reduces the porosity of cement paste by lowering the number of tiny pores (<50 nm), and the total porosity and average pore diameter decline with an increasing the content of the GC hybrid.

## 5. Conclusions

This paper prepared GC hybrid–cement composites using a GC aqueous dispersion and analyzed the effects of the GC hybrid on the early hydration process, mechanical properties, and microstructure of the cementitious composites. The results showed that the GC hybrid facilitated the early hydration process of cement paste. The 3-, 7-, and 28-day compressive strength of the cement paste with 0.10 wt% of the GC hybrid increased by 13.78%, 10.19%, and 8.40%, respectively. The microscopic analysis also revealed that the GC hybrid could reduce the porosity of cement paste and promote portlandite production. Further, the connection between the hydration products was enhanced by the unique three-dimensional spatial structure of the GC hybrid. The average microhardness of the cement paste containing 0.10 wt% of the GC hybrid increased by 29.99% compared to the blank.

## Figures and Tables

**Table 1:** The properties of the GC hybrid.

Species	Purity (%)	The Outer Diameter of CNT (nm)	Median Size (µm)
GC	>90%	50–80	3–6

**Table 2:** The mixing proportions of the cement pastes.

Specimen ID	Cement (g)	GC Hybrid (g)	Surfactant (g)	Water (g)
R	100	0	0.02	40
GC1	100	0.05	0.02	40
GC2	100	0.10	0.02	40
GC3	100	0.15	0.02	40

**Table 3:** The enhancement effect of carbon nanomaterials on the mechanical properties of the cementitious composites.

Ref	Weight Fraction	Increase in Flexural Strength (%)	Increase in ComPressive Strength (%)	Increase in Microhardness (%)
Liu et al. [17]	0.05 wt% of CNTs	8.8	4.1	10.1
Liu et al. [17]	0.05 wt% of graphene	3.3	4.3	8.9
Liu et al. [17]	0.10 wt% of CNTs	7.7	0.5	8.3
Liu et al. [17]	0.10 wt% of graphene	12.1	4.6	18.3
Lu et al. [40]	0.05 wt% of GO	8.3	6.8	-
Li et al. [19]	0.5 wt% of CNTs	25	19	-
This study	0.05 wt% of GC hybrid	6.6	12.3	22.9
This study	0.10 wt% of GC hybrid	13.1	18.4	31.8

## Data Availability

Data are available on request from the corresponding author.
